# A new ankylosaurid from the Upper Cretaceous Nemegt Formation of Mongolia and implications for paleoecology of armoured dinosaurs

**DOI:** 10.1038/s41598-021-02273-4

**Published:** 2021-11-25

**Authors:** Jin-Young Park, Yuong-Nam Lee, Yoshitsugu Kobayashi, Louis L. Jacobs, Rinchen Barsbold, Hang-Jae Lee, Namsoo Kim, Kyo-Young Song, Michael J. Polcyn

**Affiliations:** 1 School of Earth and Environmental Sciences, Seoul National University, Seoul, 08826 South Korea; 2 Hokkaido University Museum, Hokkaido University, Sapporo, Hokkaido 060-0801 Japan; 3 Roy M. Huffington Department of Earth Sciences, Southern Methodist University, Dallas, TX 75275 USA; 4 Institute of Paleontology, Mongolian Academy of Sciences, Box-46/650, Ulaanbaatar, 15160 Mongolia; 5 Korea Institute of Geoscience and Mineral Resources, Daejeon, 34123 South Korea; 6 Department of Earth System Sciences, Yonsei University, Seoul, 03722 South Korea

**Keywords:** Evolution, Palaeontology

## Abstract

A new ankylosaurid dinosaur, *Tarchia tumanovae* sp. nov., has been recovered from the Upper Cretaceous Nemegt Formation of Mongolia. It includes a well-preserved skull, dorsal, sacral, caudal vertebrae, sixteen dorsal ribs, ilia, a partial ischium, free osteoderms, and a tail club. The squamosal horns of *T. tumanovae* are divided into two layers, the external dermal layer and the underlying squamosal horn proper. The irregular ventral margin of the base of the upper dermal layer may represent a resorption surface, suggesting that the squamosal horns of some ankylosaurids underwent extreme ontogenetic remodeling. Localized pathologies on the dorsosacral ribs and the tail provide evidence of agonistic behaviour. The tail club knob asymmetry of *T. tumanovae* resulted from restricted bone growth due to tail club strikes. Furthermore, *T. tumanovae* had an anteriorly protruded shovel-shaped beak, which is a morphological character of selective feeders. Ankylosaurid diets shifted from low-level bulk feeding to selective feeding during the Baruungoyot and the Nemegt “age” (middle Campanian-lower Maastrichtian). This ankylosaurid niche shifting might have been a response to habitat change and competition with other bulk-feeding herbivores.

## Introduction

Ankylosaurid dinosaurs, one group of Ankylosauria, are quadrupedal, herbivorous, and have a heavily ornamented skull and parasagittal rows of osteoderms covering the dorsolateral surfaces of the body^1^. Their fossils have been frequently discovered from the Upper Cretaceous (upper Campanian-lower Maastrichtian) Nemegt Formation of Mongolia^2–9^. Except for the holotype of *Tarchia teresae* (PIN 3142/250), previously known specimens from this rock unit consist of only postcranial material, mostly caudal elements.

**Figure 1:**
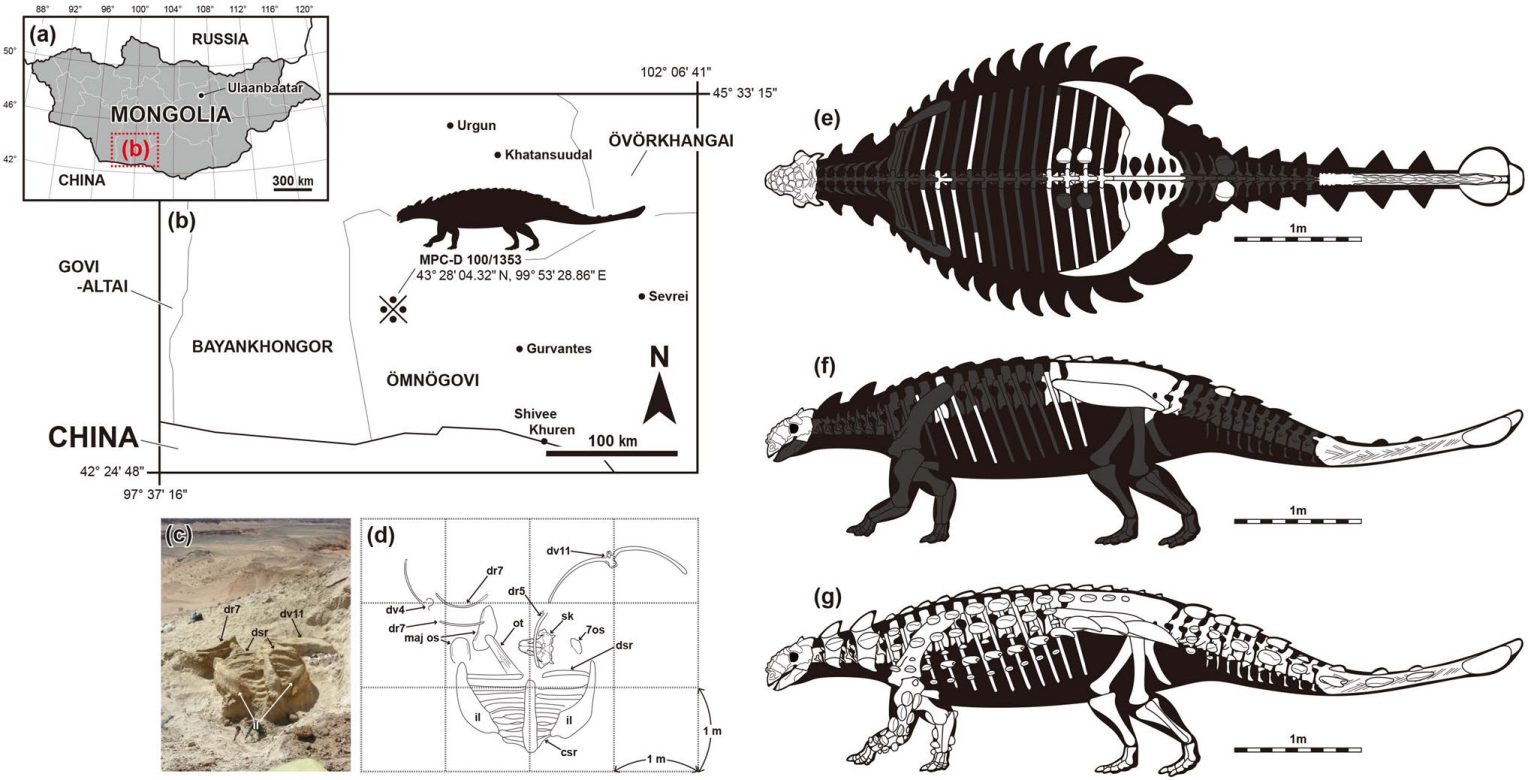
Map showing the locality where *Tarchia tumanovae* sp. nov. (MPC-D 100/1353) was discovered (**a–d**). (**a**) Map of Mongolia. (**b**) Enlarged map of the dotted lined rectangle of A marked with the fossil locality (※). (**c**) Photo of the excavation site. (**d**) Quarry map showing bone location. (**e–f**) Skeletal diagram of the specimen in dorsal (**e**) and left lateral (**f**) views (white bones represent recovered elements). (**g**) Skeletal reconstruction with dermal armour. Abbreviations: 7os, Type 7 osteoderm; csr, caudosacral vertebra; dr, dorsal rib; dsr, dorsosacral vertebra; il, ilium; maj os, major osteoderm; ot, ossified tendon; sk, skull. Adobe Illustrator CC (version 24.0.1, https://www.adobe.com/kr/products/illustrator.html) was employed to produce (**a–g**).

**Figure 2:**
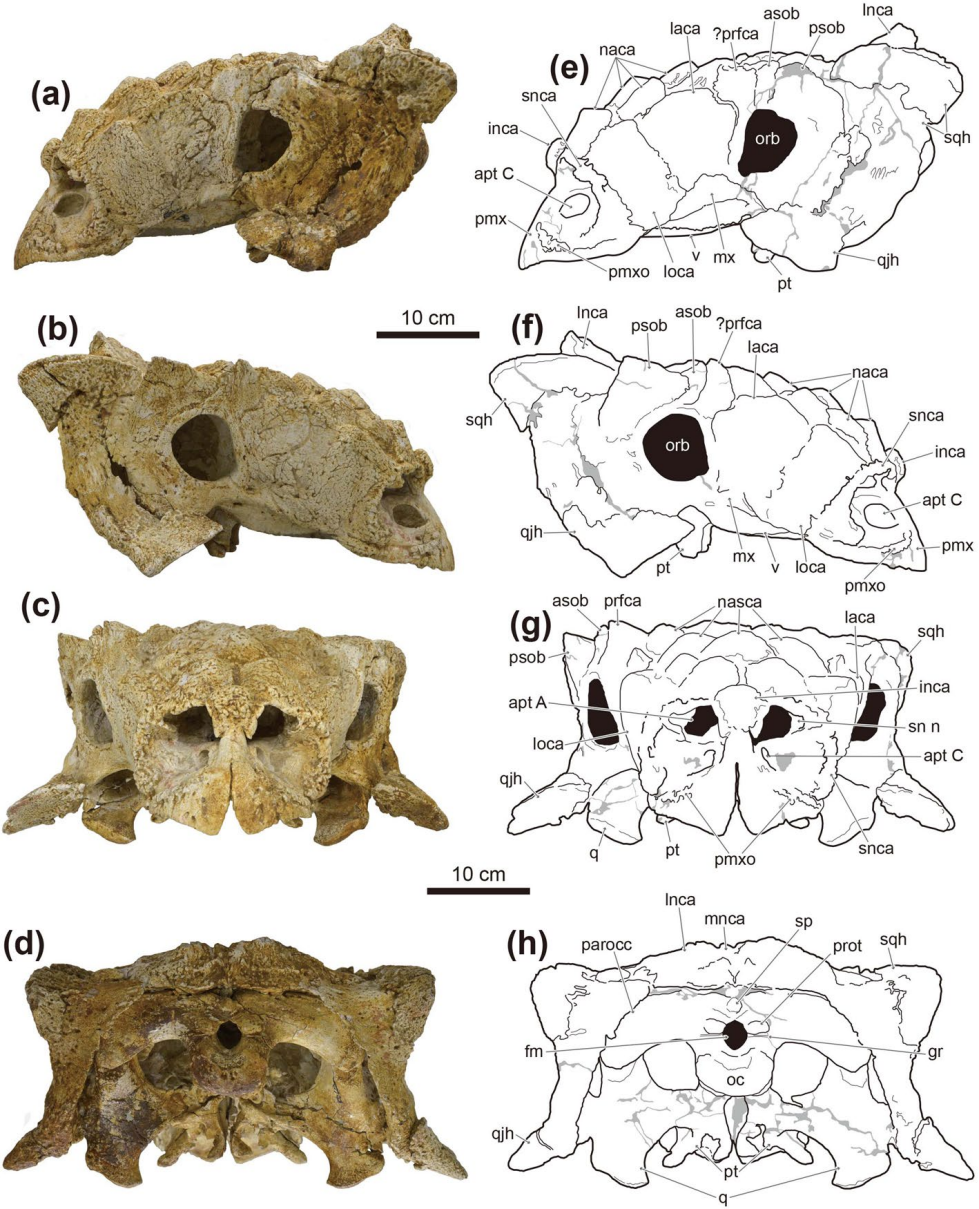
Photographs (**a**–**d**) and line drawings (**e**–**h**) of the skull of *Tarchia tumanovae* sp. nov. (MPC-D 100/1353). Photographs of the skull in (**a**) left lateral, (**b**) right lateral, (**c**) anterior, and (**d**) occipital views. Line drawings in (**e**) left lateral, (**f**) right lateral, (**g**) anterior, and (**h**) occipital views. Grey areas indicate damaged surfaces. *apt A* aperture A, *apt C* aperture C, *asob* anterior supraorbital caputegulum, *fm* foramen magnum, *gr* groove, *inca* internarial caputegulum, *laca* lacrimal caputegulum, *lnca* lateral nuchal caputegulum, *loca* loreal caputegulum, *mnca* medial nuchal caputegulum, *mx* maxilla, *naca* nasal caputegulum, *oc* occipital condyle, *orb* orbit, *parocc* paroccipital process, *pmx* premaxilla, *pmxo* premaxillary ornamentation, *prfca* prefrontal caputegulum, *prot* protuberance, *psob* posterior supraorbital caputegulum, *pt* pterygoid, *q* quadrate, qjh quadratojugal horn, *sn n* supranarial notch, *snca* supranarial caputegulum, *sp* small process between the foramen magnum and the nuchal shelf, *sqh* squamosal horn, *v* vomer. Adobe Illustrator CC (version 24.0.1, https://www.adobe.com/kr/products/illustrator.html) was employed to produce (**e**–**h**).

**Figure 3:**
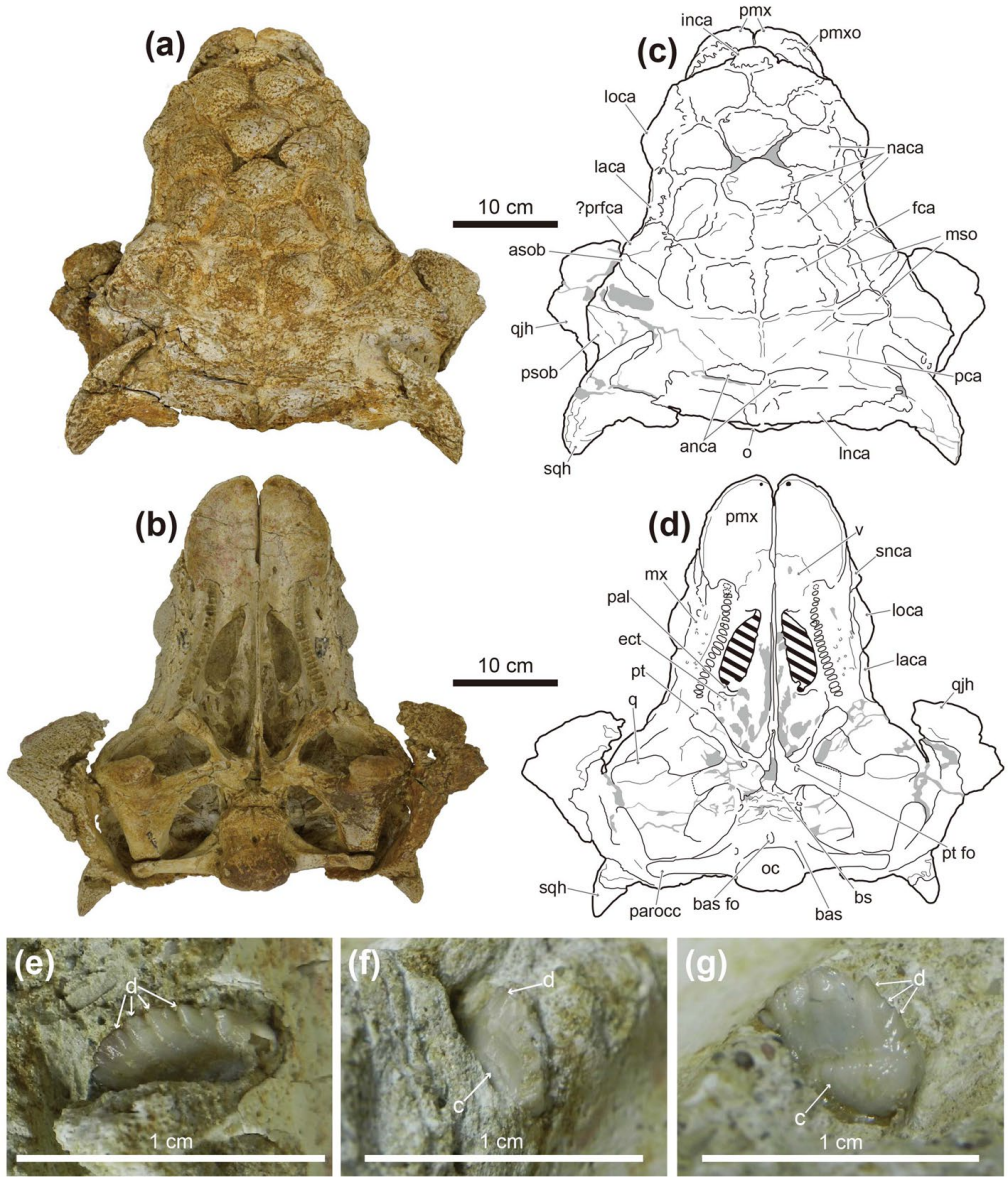
Photographs (**a** and **b**) and line drawings (**c** and **d**) of the skull, and photographs of the maxillary teeth (**e**–**g**) of *Tarchia tumanovae* sp. nov. (MPC-D 100/1353). Photographs of the skull in (**a**) dorsal and (**b**) palatal views. Line drawings in (**c**) dorsal and (**d**) palatal views. Grey areas indicate damaged surfaces, and solid diagonal lines indicate unremoved matrix. Photographs of (**e**) seventh left, (**f**) third right, and (**g**) eight right maxillary teeth in anterolabial view. *anca* anterolateral nuchal caputegulum, *asob* anterior supraorbital caputegulum, *bas* basioccipital, *bas fo* basioccipital foramen, *bs* basisphenoid, *c* cingulum, *d* denticle, *ect* ectopterygoid, *fca* frontal caputegulum, *inca* internarial caputegulum, *laca* lacrimal caputegulum, *lnca* lateral nuchal caputegulum, *loca* loreal caputegulum, *mnca* medial nuchal caputegulum, *mso* middle supraorbital caputegulae, *mx* maxilla, *naca* nasal caputegulum, *oc* occipital condyle, *pal* palatine, *parocc* paroccipital process, *pca* parietal caputegulum, *pmx* premaxilla, *pmxo* premaxillary ornamentation, *prfca* prefrontal caputegulum, *psob* posterior supraorbital caputegulum, *pt* pterygoid, *pt fo* pterygoid foramen, *q* quadrate, *qjh* quadratojugal horn, *snca* supranarial caputegulum, *sqh* squamosal horn, *v* vomer. Adobe Illustrator CC (version 24.0.1, https://www.adobe.com/kr/products/illustrator.html) was employed to produce (**c** and **d**).

**Figure 4:**
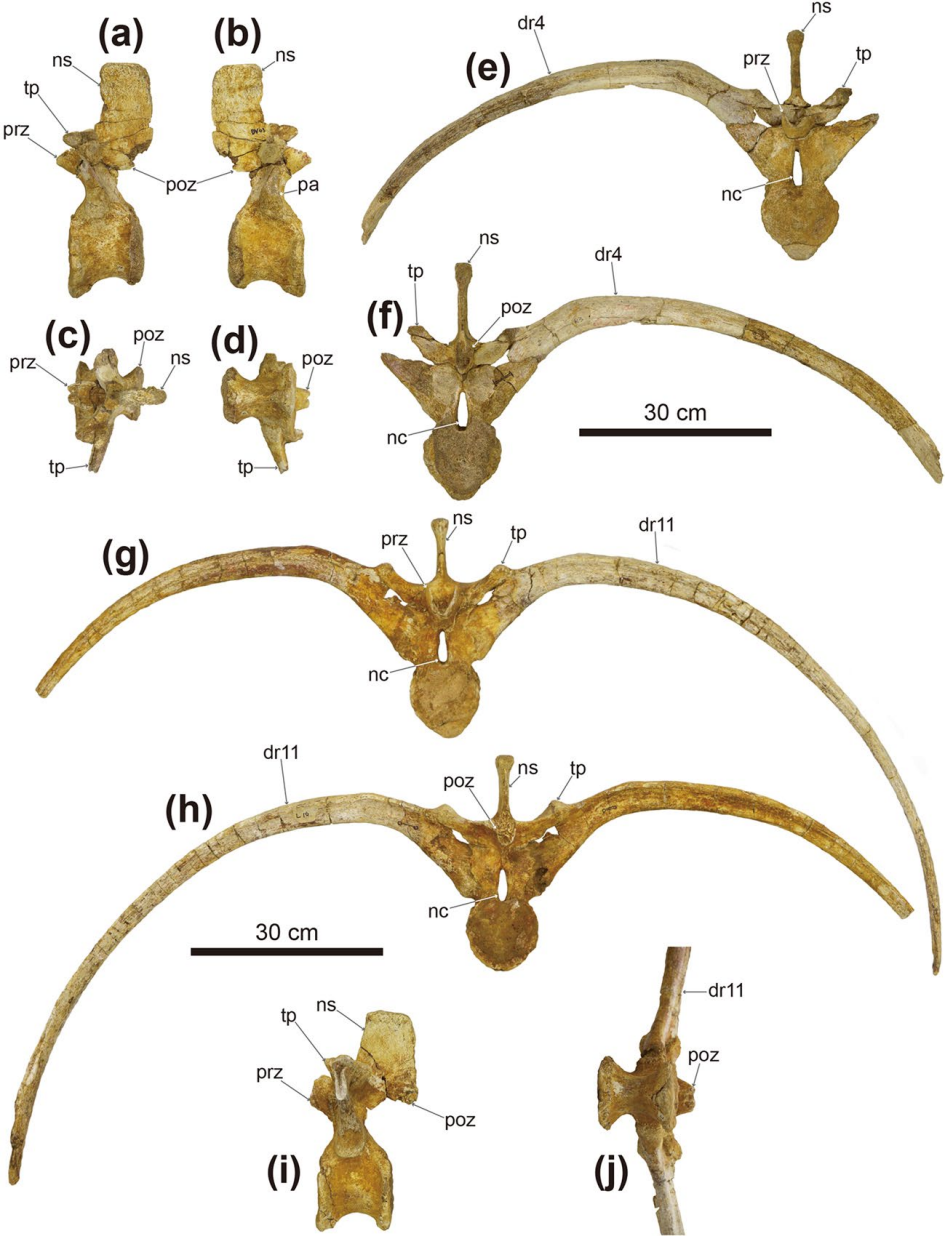
Photographs of dorsal vertebrae of *Tarchia tumanovae* sp. nov. (MPC-D 100/1353). The fourth dorsal vertebra in (**a**) left lateral, (**b**) right lateral, (**c**) dorsal, and (**d**) ventral views. Fourth dorsal vertebra with fused right rib in (**e**) anterior and (**f**) posterior views. The eleventh dorsal vertebra in (**g**) anterior, (**h**) posterior, (**i**) left lateral (with no ribs attached), and (**j**) ventral views. *dr* dorsal rib, *nc* neural canal, *ns* neural spine, *pa* parapophysis; prezygapophysis, *poz* postzygapophysis, *tp* transverse process.

**Figure 5:**
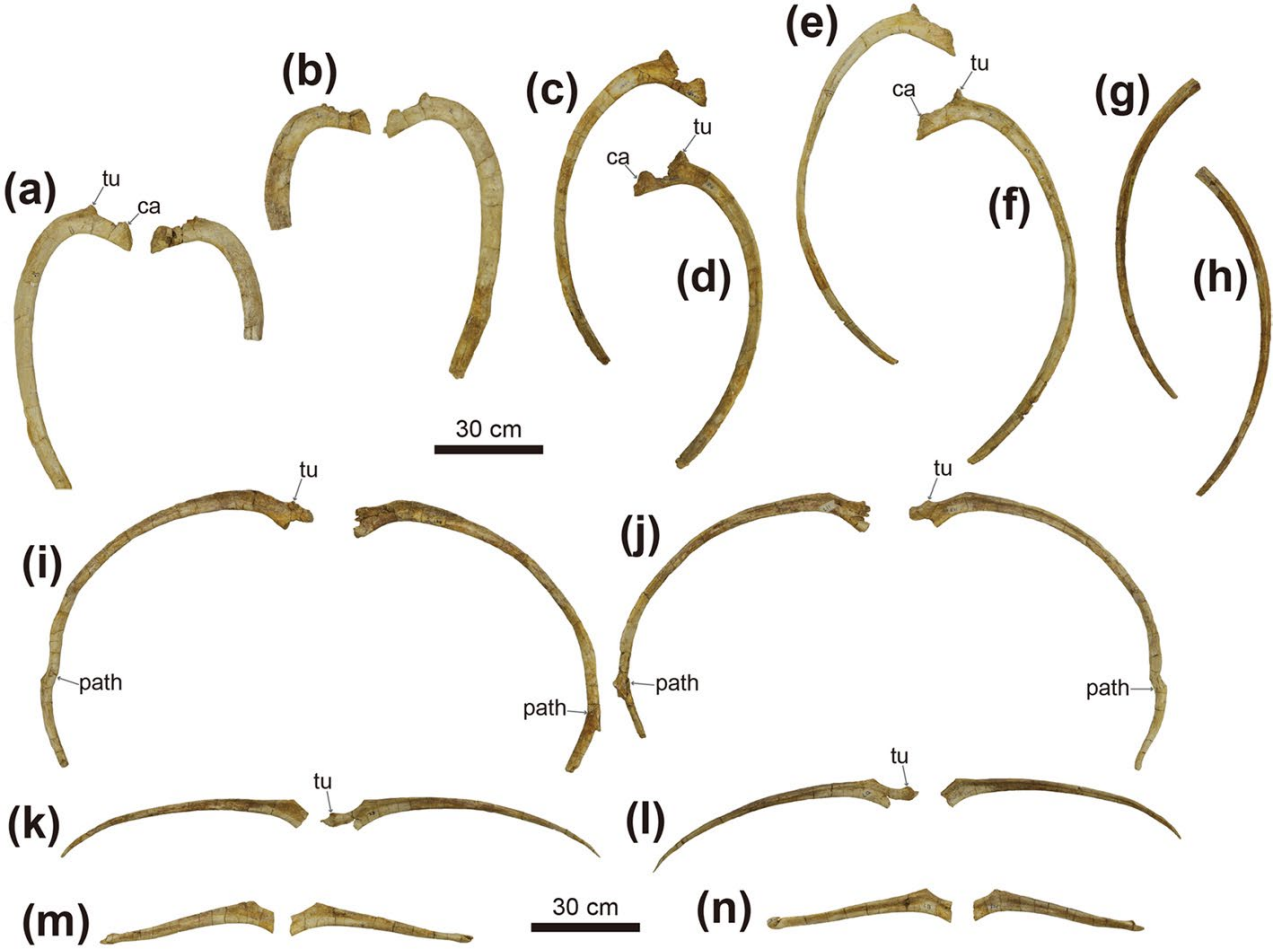
Photographs of dorsal (**a**–**h**) and dorsosacral ribs (**i**–**p**) of *Tarchia tumanovae* sp. nov. (MPC-D 100/1353). Both third dorsal ribs in (**a**) anterior and (**b**) posterior views. The fourth left dorsal rib in (**c**) anterior and (**d**) posterior views. The fifth left dorsal rib in (**e**) anterior and (**f**) posterior views. The sixth left dorsal rib in (**g**) anterior and (**h**) posterior views. Both first dorsosacral ribs in (**i**) anterior and (**j**) posterior views. Both second dorsosacral ribs in (**k**) anterior and (**l**) posterior views. Both third dorsosacral ribs in (**m**) anterior and (**n**) posterior views. *ca* capitulum, *path* pathology, *tu* tuberculum.

**Figure 6:**
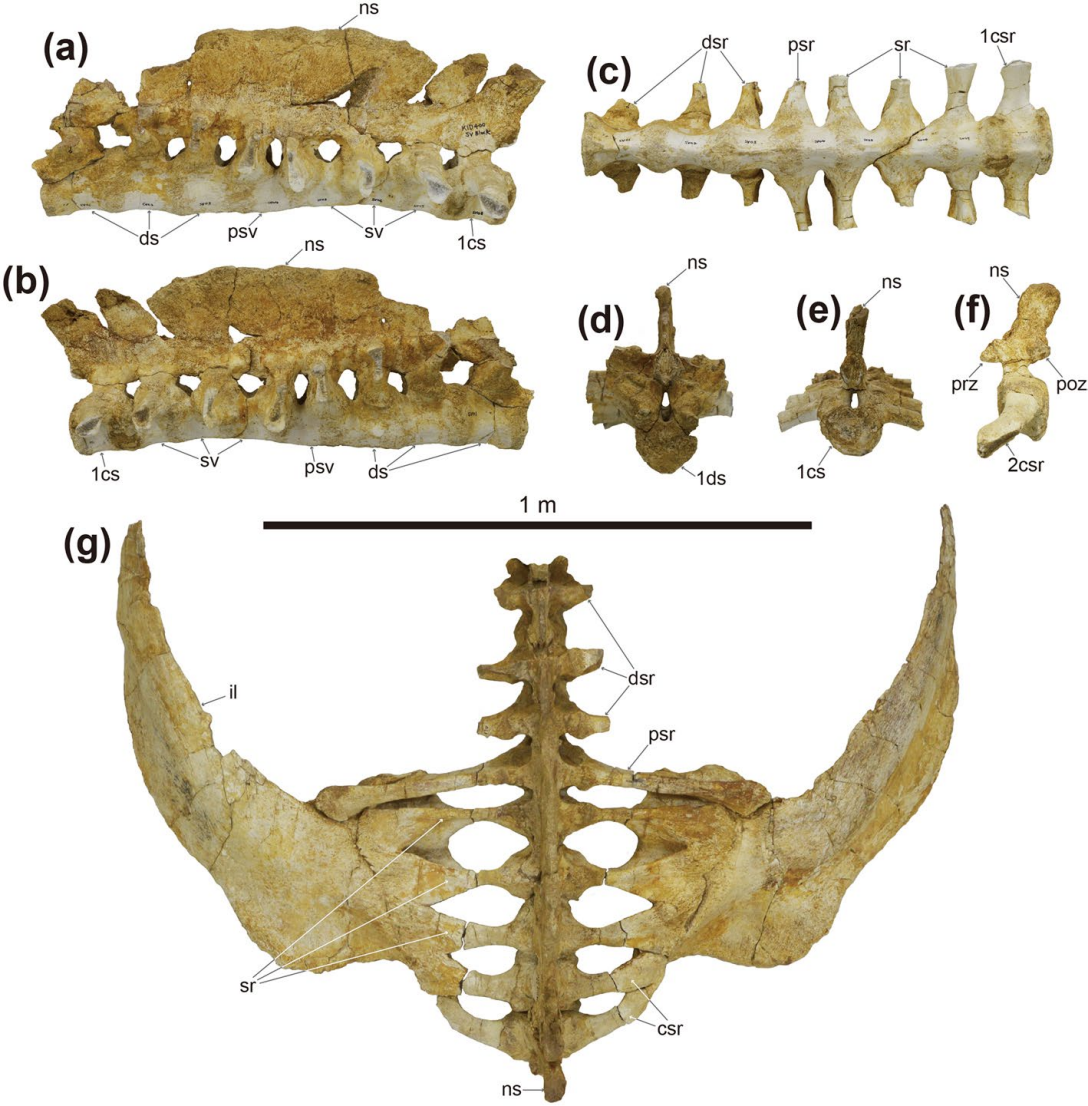
Photographs of the synsacrum (**a**–**f**), and ilia (**g**) of *Tarchia tumanovae* sp. nov. (MPC-D 100/1353). Synsacrum in (**a**) left lateral, (**b**) right lateral, (**c**) ventral, (**d**) anterior, and (**e**) posterior views. (**f**) Second caudosacral vertebra in left lateral view. (**g**) Synsacrum with the fused ilia in dorsal view. *cs* caudosacral vertebra, *csr* caudosacral rib, *ds* dorsosacral vertebra, *dsr* dorsosacral rib, *il* ilium, *ns* neural spine, *psr* parasacral rib, *psv* parasacral vertebra, *sr* sacral rib, *sv* sacral vertebra.

**Figure 7:**
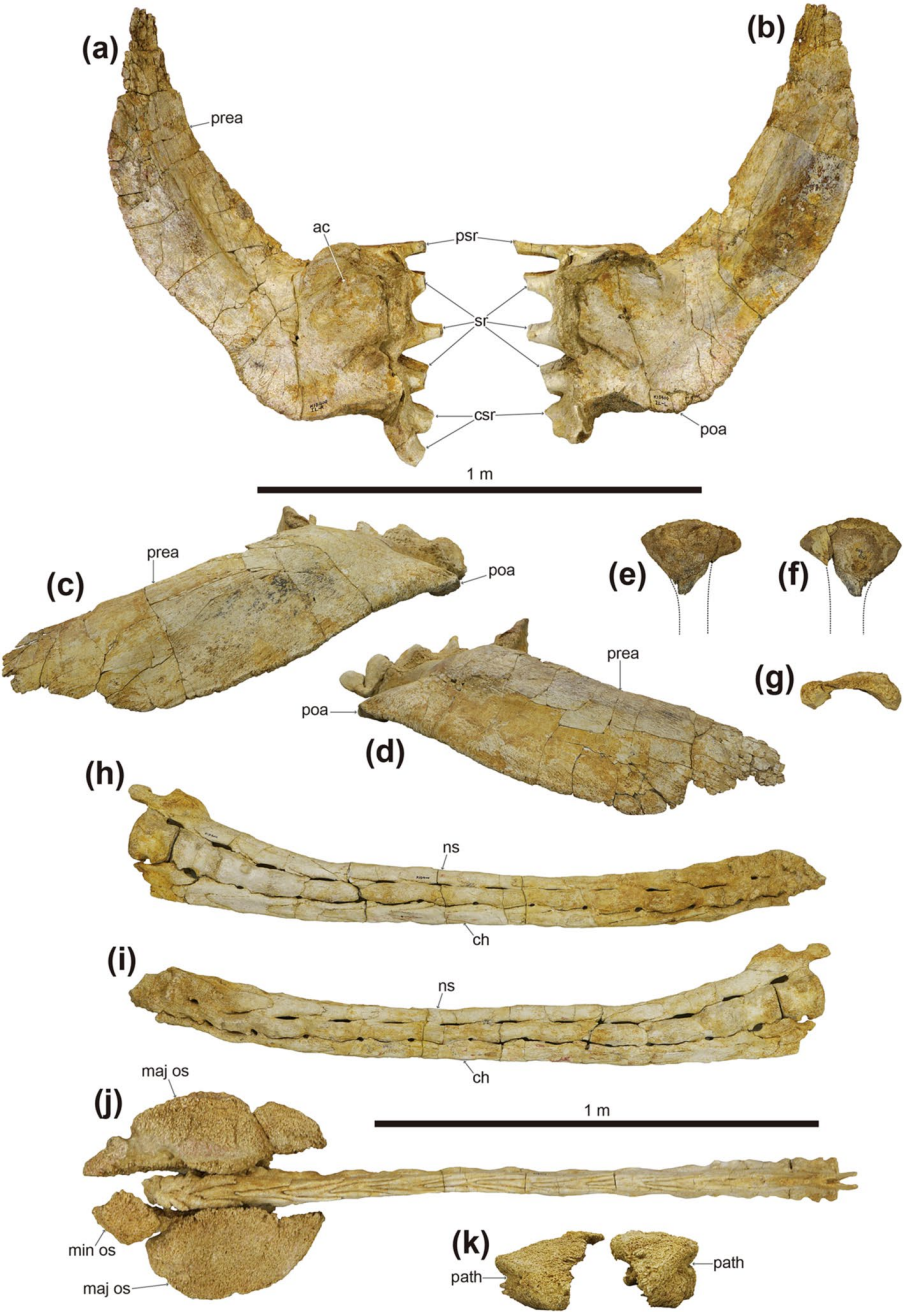
Photographs of both ilia (**a**–**d**), partial left ischium (**e**–**g**), and the tail club (**h**–**k**) of *Tarchia tumanovae* sp. nov. (MPC-D 100/1353). (**a**) Left and (**b**) right ilium in ventral view. (**c**) Right and (**d**) left ilium in lateral view. Left ischium in (**e**) lateral, (**f**) medial, and (**g**) proximal views. Tail club handle in (**h**) left lateral and (**i**) right lateral views. (**j**) Tail club handle and knob in dorsal view. (**k**) Tail club knob in proximal view. *ac* acetabulum, *ch* chevron, *csr* caudosacral rib, *maj os* major osteoderm, *min os* minor osteoderm, *ns* neural spine, *path* pathology, *poa* postacetabular process, *prea* preacetabular process, *psr* parasacral rib, *sr* sacral rib.

**Figure 8:**
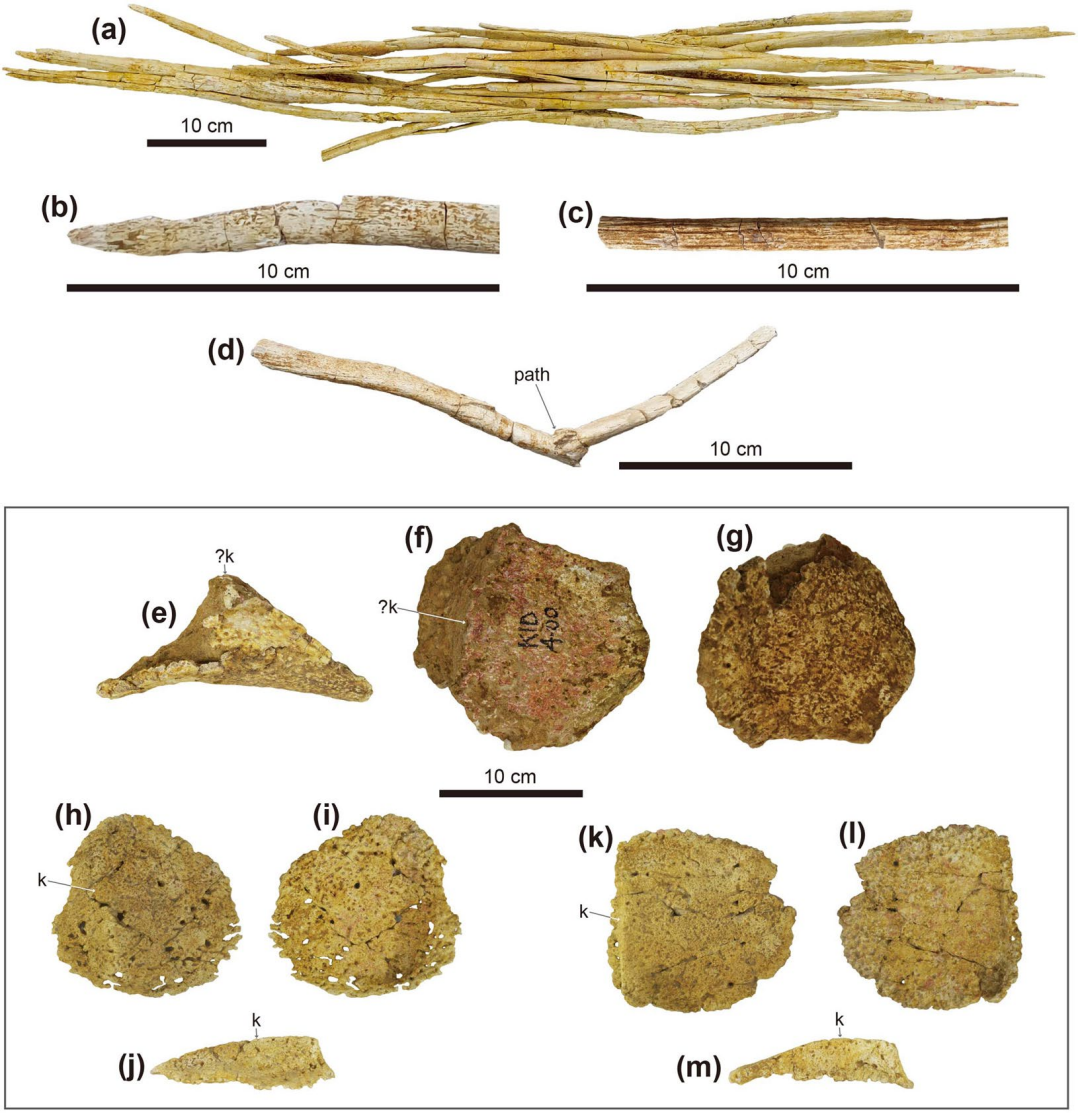
Photographs of ossified tendons from the tail knob handle (**a**–**d**) and dermal osteoderms (**e**–**m**) of *Tarchia tumanovae* sp. nov. (MPC-D 100/1353). (**a**) A bundle of ossified tendons from the tail knob handle. Close up photos of (**b**) an ossified tendon that is flattened and (**c**) a tendon that is elliptic in cross-section and has longitudinal striae. (**d**) An ossified tendon with evidence of fracture healing. Type 2 osteoderm in (**e**) anterior, (**f**) oblique dorsolateral, and (**g**) ventral views. Two Type 7 osteoderms in (**h** and **k**) dorsal, (**i** and **l**) ventral, and (**j** and **m**) lateral views. *k* keel, *path* pathology.

During the Korea-Mongolia International Dinosaur Expedition in 2008, a new skull with a partial postcranial skeleton (MPC-D 100/1353) was collected from the Nemegt Formation in Hermiin Tsav (Fig. 1). The specimen includes a well-preserved skull, dorsal, sacral, caudal vertebrae, sixteen dorsal ribs, ilia, a partial ischium, free osteoderms, and a tail club (Figs. 2, 3, 4, 5, 6, 7, 8), and turned out to be a new taxon. As a new taxon, it is named, described, and discussed herein. The new specimen provides further evidence of ontogeny, agonistic behavior, and suggestions of niche shifting in Mongolian ankylosaurids.

## Results

### Systematic paleontology

Dinosauria Owen^10^.

Ankylosauridae Brown^11^.

Ankylosaurinae Nopcsa^12^.

*Tarchia* Maryañska^13^.

**Type species.** *Tarchia kielanae* Maryañska^13^.

**Revised diagnosis.** An ankylosaurid distinguished by having the following unique set of characters (autapomorphies with an asterisk): a narrow internarial bar of the premaxillae (shared with *Tsagantegia*) (ambiguous in *Tarchia kielanae*); large, rhomboidal loreal caputegulum with a laterally extended posterior keel (shared with *Saichania*) (ambiguous in *T. kielanae*); subrectangular frontal caputegulae (shared with *Saichania*); a “neck” present at the base of the quadratojugal horn (shared with *Pinacosaurus mephistocephalus* and *Minotaurasaurus*) (ambiguous in *T. kielanae*); sigmoidal and peaked anteromedial supraorbital caputegulum*; posterolateral supraorbital caputegulum with a rounded anterior surface, and a flat, anteriorly-inclined posterior surface*; anteromedially poorly defined postorbital fossa that medially reaches the lateral nuchal caputegulae*; occiput visible in dorsal view (shared with *Minotaurasaurus* and *Zaraapelta*); foramen magnum taller than wide*. Differs from *Minotaurasaurus*, *Pinacosaurus grangeri*, *Saichania*, and *Zaraapelta* in having no postocular caputegulae (ambiguous in *T. kielanae*) and a posteroventrally oriented occipital condyle. Differs from *Minotaurasaurus*, *P. grangeri*, and *Zaraapelta* in having confluent supraorbital horns. Differs from *Minotaurasaurus* and *Saichania* in having a relatively tall braincase. Differs from *Minotaurasaurus* and *Zaraapelta* in having a long nuchal crest. Differs from *Minotaurasaurus* in having relatively long paroccipital processes that laterally reach the squamosal horns. Differs from *Saichania* in having remodeled squamosal horns and anteroposteriorly short lateral nuchal caputegulae.

*Tarchia tumanovae* sp. nov.

**Etymology.** Named in honour of Tatiana Tumanova for her contributions toward the understanding of Mongolian ankylosaurs.

**Holotype.** MPC-D 100/1353 (Figs. 1, 2, 3, 4, 5, 6, 7, 8), a well-preserved skull, dorsal, sacral, caudal vertebrae, sixteen dorsal ribs, ilia, a partial ischium, free osteoderms, and tail club.

**Locality and horizon.** Upper Cretaceous (upper Campanian-lower Maastrichtian) Nemegt Formation, Hermiin Tsav, southern Gobi Desert, Mongolia.

**Diagnosis.** An ankylosaurid distinguished by having the following unique set of characters: a single relatively bulbous internarial caputegulum that does not reach the rostral tip of beak*; a nasofrontal sagittal furrow with a weak Z-shaped offset (shared with *Tarchia kielanae*); lateral nuchal caputegulae taller laterally than medially (shared with *Saichania*); vomerine keel extends below the alveolar ridge (shared with *Saichania*). Differs from *Minotaurasaurus*, *Pinacosaurus grangeri*, *T. kielanae*, *T. teresae*, and *Zaraapelta* in having a moderate-sized basioccipital foramen. Differs from *Minotaurasaurus*, *P. grangeri*, *Saichania*, and *Zaraapelta* in having no postocular caputegulae and a posteroventrally oriented occipital condyle. Differs from *Minotaurasaurus*, *P. grangeri*, *T. teresae*, and *Zaraapelta* in having an anteriorly situated quadrate-quadratojugal region. Differs from *Minotaurasaurus*, *P. grangeri*, and *Zaraapelta* in having confluent supraorbital horns. Differs from *P. grangeri*, *Saichania*, and *Zaraapelta* in having a tall foramen magnum. Differs from *Saichania*, *T. kielanae*, and *Zaraapelta* in having unfused quadrate to the exoccipital area. Differs from *Minotaurasaurus* and *Saichania* in having a relatively tall braincase. Differs from *Minotaurasaurus* and *Zaraapelta* in having subrectangular frontal caputegulae and a long nuchal crest. Differs from *Minotaurasaurus* in having narrow narial caputegulae and long paroccipital processes that laterally reach the squamosal horns. Differs from *Saichania* in having remodeled squamosal horns, anteroposteriorly short lateral nuchal caputegulae, and occiput visible in dorsal view. Differs from *T. teresae* by having an interpterygoid vacuity visible in occipital view.

**Description.** The shape of the skull is trapezoidal and broader than long in dorsal view (Fig. 3, see Supplementary Information S1 for measurements). All caputegulae are pitted externally. The neuroanatomy of MPC-D 100/1353 was fully described before by Paulina-Carabajal et al.^9^.

The premaxillae are fused dorsally, but the palatal surfaces are separate (Figs. 2, 3). The rostral tip is protruded anteroventrally, and a premaxillary notch is present. The narrow internarial bar is oriented posterodorsally. A thin, rugose ossification is present on each premaxilla below the external naris. The anterior margin of the premaxillary ornamentation is convex, whereas the posterior margin is concave. The palatal surface of the premaxillae is shovel-like with a round anterior boundary. No premaxillary teeth are present. The subcircular external nares face anteriorly. The entrance to the airway (aperture A, *sensu*^14^) is large and subcircular. The airway is filled with a matrix. A supranarial notch is present on the medial wall of the maxillary region, lateral to the entrance to the airway. A short, medioventrally sharp intranasal process is present beneath the entrance of this aperture. A larger oval dorsolaterally-facing paranasal aperture (aperture C) is situated on the ventral wall of the external nares behind the internasal bar. The external nares are rimmed dorsomedially, dorsally, and laterally by supranarial caputegulae (*sensu*^15^). The dorsomedial and dorsal portion of the supranarial caputegulum is thin, whereas the wide ventrolateral part gives the caputegulum a boot-like appearance in lateral view. The anterior end of the nasals contacts the internasal bar anteromedially. A single medium-sized bulbous internarial caputegulum is situated above the contact between the internarial bar of the premaxillae and the anterior nasals and between the thin medial portion of the two supranarial caputegulae. Behind the internarial caputegulum, eleven pyramidal nasal caputegulae are present. Most of these caputegulae are large and surrounded by a broad sulcus. The most medioposteriorly positioned pair are transversely oriented, pyramidal, and rectangular, similar to the frontal caputegulae.

The maxillae are anteroposteriorly elongate, extending to below the orbits. In lateral view, the anterior portion of the maxillae is covered by the loreal caputegulum, whereas the posterior portion is exposed. In palatal view, the convex maxillary tooth row is situated medial to the buccal emargination. Nineteen alveoli are present in each maxilla. A single loreal caputegulum is present on each side. The loreal caputegulum is large, rhomboid, and has a posterior keel that posterolaterally juts out. The lacrimal caputegulum is large and flat laterally and has a keeled edge on the dorsal margin. A single medium-sized possible prefrontal caputegulum is present on each side, forming the lateral margin of the skull. The left possible prefrontal caputegulum is damaged, but the right is well preserved. This caputegulum is keeled dorsally and has a lateroposteriorly pointing apex in close contact with the anterior supraorbital caputegulum.

Paired, transversely oriented frontal caputegulae are pyramidal and rectangular (Fig. 3a,c). The nasofrontal sagittal furrow has a weak Z-shaped offset as in ZPAL MgD I/111 (holotype of *T. kielanae*). Although the parietal caputegulae are poorly defined, three shallow dorsal furrows diverge anteroposteriorly from each other at an angle of about 35°. Two medial supraorbital caputegulae are present on the right, whereas only the anteromedial one is preserved on the left. The longitudinally oriented anteromedial caputegulum is sigmoidal. The transversally oriented posterolateral caputegulum has a rounded anterior surface, a flat, anteriorly-inclined posterior surface. The left supraorbital caputegulae have damaged apices, whereas the right ones are well preserved with a distinct apex. The anterior supraorbital caputegulum is narrow and keeled, with an anterolaterally directed apex. The posterior supraorbital caputegulum is laterally pointed and keeled dorsally and is about five times larger than the anterior supraorbital caputegulum. The orbits have a posteriorly thick orbital rim and face anterolaterally. The quadratojugal horns are triangular and project ventrolaterally (Figs. 2, 3). A quadratojugal “neck” (*sensu*^16^) is present at the base of the horns. Postocular caputegulae are absent. The squamosal horns are pyramidal and posteriorly recurved (Figs. 3a,c). These horns are divided into the upper external layer of the squamosal horn and the underlying squamosal horn proper (*sensu*^17^). The former is dorsolaterally keeled with a longitudinal furrow present on the posterior half of the keel. The narrow, sharp, and medially curved anterior portion of the caputegulae lies in a broad, deep postorbital fossa posterior to the supraorbital. The anterior tip of the right horn was slightly broken sometime after CT scanning of the braincase by Paulina-Carabajal et al.^9^. A narrow, deep sulcus separates the irregular ventral margin of the base of the external layer of the squamosal horn and the underlying squamosal horn proper. The surface of the external dermal layer is pitted, whereas the squamosal horn proper has a granular texture. The nuchal shelf is dorsally uplifted and does not overhang the outer rim of the skull. Two nuchal caputegulae are present on each side. Both anterior and lateral nuchal caputegulae are elongate and transversely positioned. However, the lateral nuchal caputegulae are about four times larger than the anterior nuchal caputegulae, forming the posterior margin of the cranium. In occipital view, the nuchal shelf is not fused with the supraoccipital and paroccipital processes (Fig. 2d,h).

The rostral extension of the vomer is dorsoventrally thin, splayed, and fused with the posteromedial region of the premaxillae (Fig. 3b,d). The osseous nasal septum (*sensu*^18^) extends dorsally but does not meet the skull roof. The vomerine keel ends ventral to the alveolar ridge. The palatine extends posteromedially from the maxilla and gently projects dorsally, forming a posteroventral secondary palate (*sensu*^18^). The ectopterygoid is small and wedge-like. The pterygoid has a vertical anterior surface with a foramen pierced through the central body. The pterygoid flange projects anterolaterally and contacts the dorsally positioned ectopterygoid. The quadrate ramus contacts the posterolaterally positioned quadrate. The posterolateral edge of the quadrate ramus is damaged on both sides. The posteromedial margin of the main pterygoid body is not fused with the basipterygoid processes of the basisphenoid. The basipterygoid processes are divided from each other. An interpterygoid vacuity is present between the paired pterygoids.

In palatal view, the contact between the basisphenoid and the basioccipital forms a rugose transverse ridge (Fig. 3b,d). A basioccipital foramen is present on the convex ventral surface of the basioccipital. The basioccipital and the exoccipitals are entirely fused and form a reniform occipital condyle oriented posteroventrally. The ovoid foramen magnum is taller than wide (Fig. 2d,h). A small hill-like process lies between the foramen magnum and the nuchal shelf. A horizontal groove is present below the paired exoccipital protuberances. The lateral terminus of the paroccipital process is long but not fused to the quadrate reaching laterally to the squamosal horns. The transversely broad quadrates are inclined anteroventrally toward the distal articular condyles in lateral view. The medial condyle of the quadrate is larger than the lateral condyle.

Only the third and eighth right maxillary teeth and the seventh left maxillary tooth are preserved (Fig. 3e–g). Although these teeth are partially embedded within the sockets, up to eleven marginal denticles can be observed. Shallow vertical grooves are present between the denticles, and a shelf-like labial cingulum is also present.

Two isolated dorsal vertebrae with fused ribs (Fig. 4), probably presenting the fourth and eleventh dorsal vertebrae based on the length of the ribs, are preserved. Only the right rib is preserved on the fourth dorsal vertebra (Fig. 4e,f), whereas both ribs are present on the eleventh (Fig. 4g,h,j). These vertebrae have amphiplatyan spool-shaped centra, similar in length and height, and laterally constricted. A medial ridge is present on the ventral surface of the centrum. The tall and narrow neural arches are centrally located on the dorsal centra. Accordingly, the neural canals are dorsoventrally tall and laterally narrow. The transverse processes are angled about 45° dorsolaterally. The dorsally directed prezygapophyses extend beyond the anterior margin of the centrum. The postzygapophyses are directed ventrally and smaller than the prezygapophyses. Including the three ribs that are fused to the free dorsal vertebrae, a total of eight free dorsal ribs are preserved (Figs. 4, 5). These ribs have an anteroposteriorly expanded, flat dorsal surface with a dorsoventrally deep head. The rib is T-shaped in a proximal cross-section. The anterior dorsal ribs, especially the second dorsal ribs, are mediolaterally broader than the posterior ribs. The articulated dorsal ribs are fused to the ventral side of the transverse processes. The fused dorsal ribs arc outward, resulting in a barrel-like trunk.

The synsacrum is well preserved and includes three dorsosacrals, one parasacral, three sacrals, and two caudosacral vertebrae (Fig. 6). These vertebrae are fused along the centra and the neural spines. The centra of these vertebrae are spool-shaped and laterally constricted. A single medial ridge is present on the ventral surface of the centra of the dorsosacrals and the parasacral. Two medial ridges are present on the first and second sacrals and both caudosacrals. The neural arches of the dorsosacrals are centrally located dorsal to the centra, whereas the neural arches of the parasacral and sacrals are shifted anteriorly, projecting beyond the anterior margin of the centra. The neural arches of the caudosacrals are also shifted anteriorly but not projected as much as in the parasacral and sacrals. The fused, laterally compressed neural spines are directed posterodorsally. All dorsosacral, parasacral, sacral, and caudosacral ribs are fused to the vertebrae, although the dorsosacral ribs were separated during preparation. The first pair of dorsosacral ribs are arced similar to the posterior dorsal ribs, whereas the second and third pairs are rod-like and much shorter in length. Bone healing is observed on both sides of the distal portion of the first dorsosacral ribs (Fig. 5i,j). In dorsal view, the ribs become shorter in mediolateral length from the parasacral to the second caudosacral. The parasacral ribs are slightly projected posterolaterally and contact the ilium. The sacral ribs are projected laterally, whereas the caudosacral ribs are projected anterolaterally. All sacral and caudosacral ribs are fused to the ilium. In lateral view, the ribs from the parasacral to caudosacrals project at a lower angle. The parasacral and the first sacral ribs have anteroposteriorly expanded dorsal and ventral surfaces, which give the ribs an I-shaped proximal cross-section (Fig. 6a,b). The cross-section of the proximal ribs becomes trapezoid from the second sacral to the second caudosacral (Fig. 6a,b).

Fourteen caudal vertebrae are fused into the handle (*sensu*^19^) of the tail club (Fig. 7h–j). The handle is slightly curved in a posterodorsal fashion. The first to second handle vertebrae are spool-shaped, similar in length and height, and laterally compressed. The centra are elongated from the third back, except the fourteenth handle vertebra is short and knob-like. The low neural spines are blade-like and decrease in height posteriorly. The paddle-like prezygapophyses project beyond the anterior margin of the centra and diverge at an angle of about 20º in dorsal view. These interlock with the distally elongate, wedge-like postzygapophyses of the adjacent vertebra. The small nub-like transverse processes are only present on the first to third handle vertebrae. The chevrons are elongate, blade-like, and fused onto the ventral surface of the centra. Well-preserved ossified tendons were uncovered along the lateral surfaces of the tail handle (Fig. 8a–d). These tendons are arranged in an imbricating pattern. Most of these tendons are flattened and periodically bifurcate into as many as four branches. Some of the tendons are elliptic in cross-section and have longitudinal striae on the external surfaces. A poorly healed pathology is preserved on one tendon. This tendon was fractured in the middle portion and fused at an angle of about 125º.

The ilia diverge at an angle of 27º from the body midline (Fig. 6). The long and blade-like preacetabular processes extend anterolaterally (Figs. 6g, 7a–d). The closed acetabulum is level with the medially positioned first and second sacral ribs. The short buttress-like postacetabular processes extend posterolaterally from the acetabulum. Only the proximal portion of the left ischium is preserved (Fig. 8e–g). The proximal margin of the ischium is convex.

No cervical half-rings were preserved. A single isolated Type 2 osteoderm (*sensu*^6^) is preserved (Fig. 8e–g). The external surfaces are damaged, whereas the medial surface is well preserved and smooth. It is polygonal in dorsal view, sharply keeled dorsally, and thin-walled. The surface texture of this osteoderm is pitted. Two isolated Type 7 osteoderms are sub-circular, dorsoventrally flattened, with a keel near one edge that tapers in height anteriorly (Fig. 8h–m). They are thin-walled, rugose externally, and have a slightly concave ventral surface. The distal end of the tail is enveloped with two major and one minor osteoderms, forming a tail club knob (Fig. 7j). The length and width of the tail club knob are nearly equal. The major osteoderms are hemispherical in dorsal view and dorsolaterally keeled; the right is slightly larger than the left. Pathological grooves are present along the lateral surfaces of the two major osteoderms (Fig. 7k). The minor osteoderm is a rhomboidal shape in dorsal view. The surface texture of the tail club knob is pitted, rugose, and spongy.

### Phylogenetic analysis

**Figure 9:**
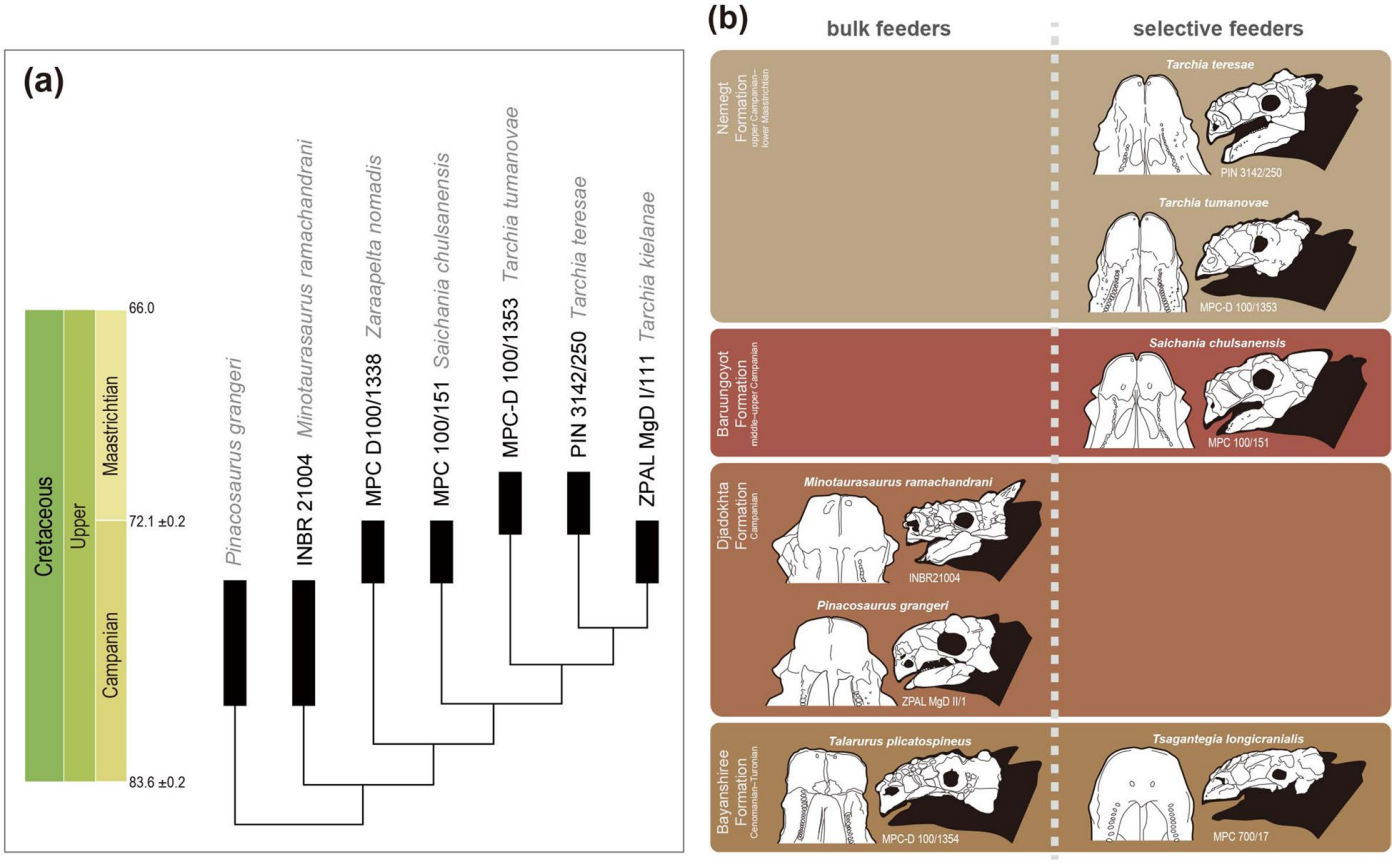
(**a**) The single most-parsimonious tree produced by phylogenetic analysis, using implied character weighting with k-value of 3. (**b**) Skull (in left lateral view) and beak (in palatal view) comparisons of Mongolian ankylosaurines and their dietary categories through time. Adobe Illustrator CC (version 24.0.1, https://www.adobe.com/kr/products/illustrator.html) was employed to produce (**a** and **b**).

The phylogenetic analysis resulted in a single most parsimonious tree (tree length = 32 steps, consistency index = 0.813, and retention index = 0.769) (Fig. 9a). *T. tumanovae* is a sister to the clade that includes *T. kielanae* and *T. teresae*. *T. kielanae* and *T. teresae* share only one synapomorphy: moderate-sized basioccipital foramen (character 10: state 1). The three *Tarchia* species shares five synapomorphies: a “neck” present at the base of the quadratojugal horn (7:1) (ambiguous in *T. kielanae*); tall foramen magnum (9:1); tall braincase (15:1); posteroventrally oriented occipital condyle (17:1); no postocular caputegulae (21:0) (ambiguous in *T. kielanae*). *Saichania*, *Zaraapelta,* and *Minotaurasaurus* were recovered as successive outgroups to the clade containing three *Tarchia* species.

## Discussion

The squamosal horns of MPC-D 100/1353 are divided into the external layer of the squamosal horn and the underlying squamosal horn propers (Fig. 2a,b,e,f). These two layers are tightly fused in the holotype of *Zaraapelta* (MPC-D 100/1338)^17^. In the holotype of *Minotaurasaurus* (INBR 21004), only the anterior end of the original osteodermal horn is present above the squamosal horn proper^20^, whereas in the holotype of *T. teresae* (PIN 3142/250), the external layers are partially preserved^4,8^. Arbour et al.^17^ hypothesized that the external dermal layer might have fused to the squamosal horn proper during ontogeny, based on MPC-D 100/1338. However, Penkalski and Tumanova^8^ suggested that MPC-D 100/1338 is immature, and the external layer might have resorbed during ontogeny, disappearing entirely in skeletally mature individuals. The divided state and shape of the squamosal horns in MPC-D 100/1353 is somewhat between MPC-D 100/1338 and INBR 21,004. Moreover, the irregular ventral margin of the base of the external dermal layer in MPC-D 100/1353 may represent resorption. If this is the case, ankylosaurines underwent extreme ontogenetic remodeling of the squamosal horns, as Penkalski and Tumanova^8^ proposed. Nonetheless, histological analysis of ankylosaurine specimens is needed to support this hypothesis further.

Evidence of fracture healing can be observed on both sides of the first dorsosacral ribs of MPC-D 100/1353, in the anterolateral part of the pelvic area (Fig. 5i,j). Arbour et al.^21^ suggested that the localized injuries on the pelvic area in ankylosaurines are likely caused by intraspecific combat inflicted by the tail club knob. This idea was supported by the fact that there was no relationship between tail club knob size and predator body mass, and concentrated pathologies observed in free caudal vertebrae, tail club knobs, and pelvic osteoderms confined to mature individuals^21,22^. A poorly healed ossified tendon on the tail knob handle is present in the holotype of *T. tumanovae* (Fig. 8d), which is a possible injury due to active tail use during combat.

The tail club knob of the holotype also has pathologies. Grooves are present along each lateral surface of the two major osteoderms (Fig. 7k). Moreover, the tail club knob is asymmetric in dorsal view, the left major osteoderm being shorter in mediolateral width than the right (Fig. 7j). Similar asymmetric bone growth has been observed on the postorbital horn of a male Dall sheep (*Ovis dalli dalli*), which impact each other with their heads at intraspecific combat^23^. As in the horn asymmetry in Dall sheep, the reduced size of the left major osteoderm in MPC-D 100/1353 could result from tail club strikes. Asymmetry of the tail club knob was noticed in three ankylosaur specimens from North American, ROM 788 (*Platypelta*), ROM 75860 (holotype of *Zuul*), and UALVP 16247 (Ankylosauridae indet.)^22,24,25^. UALVP 16247 is similar to MPC-D 100/1353 by having the left major osteoderm smaller in volume than the right^24^. In ROM 788 and ROM 75860, however, the left major osteoderm is larger than the right^22,25^. Modern African elephants (*Loxodonta*) show tusk asymmetry due to side preferences in tusk use in stripping bark, digging root, and during agonist interactions^26–28^. Comparable to the tusk asymmetry in African elephants, tail asymmetry may relate to side preferences in tail use among ankylosaurine taxa or individuals. Pathologies found on the pelvic area and tail of MPC-D 100/1353 provide additional evidence of agonistic behaviour in ankylosaurines. Laterally wide trunks of ankylosaurines could have protected vital organs from being ruptured during conspecific tail club strikes^29^.

Sub-rectangular broad muzzles are a morphological character of low-level bulk feeders, whereas anteriorly protruded shovel-shaped muzzles are selective feeders in ankylosaurines^30–32^. Similar dietary adaptations based on rostral shape are also known in mammalian herbivores, such as ungulates and ground sloths^33–36^. Based on these examples, both *Tarchia* species from the Nemegt Formation were probably selective feeders (Fig. 9b).

Bulk feeding ankylosaurines were present before the Baruungoyot and the Nemegt “age” (middle Campanian–lower Maastrichtian) based on known skull specimens (Fig. 9b). On the other hand, all ankylosaurines from the Nemegt and the Baruungoyot formations were adapted for selective feeding. These dietary shifts in ankylosaurines probably relate to habitat change, the shift from semi-arid (Bayanshiree Formation) and arid (Djadokhta and Baruungoyot formations) to more humid climates (Nemegt Formation)^31,37^. Climate-driven habitat change alters the plant communities in the environment^38^, and niche shifting in ankylosaurids might have responded to this. Recently, Jerzykiewicz et al.^39^ proposed that Djadokhta, Baruungoyot, and Nemegt Formations are coeval, and the Nemegt Gobi Basin can be visualized as an ephemeral lake surrounded by semi-arid alluvial plains and arid dune fields. If this is the case, the dietary difference between these Mongolian taxa may result from habitat differentiation within the same basin.

*T. teresae* and *T. tumanovae* coexisted with other megaherbivores, such as saurolophine hadrosaurids (*Barsboldia*^40^ and *Saurolophus angustirostris*^41^), ornithomimosaurs (*Deinocheirus*^42,43^), sauropods (*Nemegtosaurus*^44^ and *Opisthocoelicaudia*^45^), and therizinosaurs (*Therizinosaurus*^46^). Among these herbivores, the derived hadrosaurids are bulk feeders based on rostral morphology and dental microwear^47,48^. Recent phylogenic and biogeographic analyses suggest that saurolophine hadrosaurids immigrated from North America to Mongolia (Central Asia) in post-Djadokhta “age” (Campanian)^49,50^, and this record is concordant with the niche shift in Mongolian ankylosaurs. The invasion of new bulk-feeding dinosaurs, which caused interspecific competition for limited resources, possibly drove selection pressure on the diets of ankylosaurs.

## Methods

Fossil preparation of the studied specimen (MPC-D 100/1353) was done at a laboratory in Hwaseong City of South Korea in 2012. The specimen was returned to Mongolia in 2016 and is now permanently held in the Institute of Paleontology in Ulaanbaatar, Mongolia. All measurements were taken using a measuring tape and a digital caliper. Comparisons to other ankylosaurid taxa were made by examining some specimens in the Institute of Paleontology, Mongolian Academy of Sciences, Mongolia, or were extracted from published literature. The term ‘caputegulum’ (*sensu*^15^) was used to refer to the cranial ornamentation of ankylosaurs. Osteoderm types correspond to the terms used by Arbour et al.^6^.

For the phylogeny of the new *T. tumanovae*, the character list and data matrix used in this study was modified from that of Penkalski and Tumanova^8^ (Supplementary Data S2). The modifications include the following: modified one character (5); added two new characters (22 and 23); revised a few character states of *Pinacosaurus grangeri* (character state 5:0 to 15:1) and PIN 3142/250 (holotype of *Tarchia teresae*) (5:? to 5:1, and 6:0 to 6:?). Including *T. tumanovae*, seven taxa with 23 characters (Supplementary Data S3) were analyzed in TNT (Tree Analysis Using New Technology) version 1.1^51^. A traditional search was performed, using implied character weighting and a k-value of 3.

## Supplementary Information

Supplementary Information.

## Abbreviations

INBR: Victor Valley Museum, California, USA
MPC: Mongolian Paleontological Institute, Mongolian Academy of Sciences, Ulaanbaatar, Mongolia
PIN: Paleontological Institute, Russian Academy of Sciences, Moscow, Russia
ROM: Royal Ontario Museum, Toronto, Ontario, Canada
UALVP: The University of Alberta Laboratory for Vertebrate Paleontology, Edmonton, Alberta, Canada
ZPAL: Zaklad Paleobiologii (Institute of Paleobiology), Polish Academy of Sciences, Warsaw, Poland
