# Supplementary material for: A new ankylosaurid from the Upper Cretaceous Nemegt Formation of Mongolia and implications for paleoecology of armoured dinosaurs
Source: Sci Rep. 2021 Nov 25;11:22928. doi: 10.1038/s41598-021-02273-4 (PMC8616956; doi:10.1038/s41598-021-02273-4)
Supplement: Supplementary file 1 — Supplementary Information. [file 41598_2021_2273_MOESM1_ESM.docx]

**Supplementary information**

**A new ankylosaurid from the Upper Cretaceous Nemegt Formation of Mongolia and implications for paleoecology of armoured dinosaurs**

Jin-Young Park^1^, Yuong-Nam Lee^1,*^, Yoshitsugu Kobayashi^2^, Louis L. Jacobs^3^, Rinchen Barsbold^4^, Hang-Jae Lee^5^, Namsoo Kim^6^, Kyo-Young Song^5^, Michael J. Polcyn^3^

^1^ School of Earth and Environmental Sciences, Seoul National University, Seoul 08826, South Korea

^2^ Hokkaido University Museum, Hokkaido University, Sapporo, Hokkaido 060-0801, Japan

^3^ Roy M. Huffington Department of Earth Sciences, Southern Methodist University, Dallas, Texas 75275, USA.

^4^ Institute of Paleontology, Mongolian Academy of Sciences, Box-46/650, Ulaanbaatar 15160, Mongolia

^5^ Korea Institute of Geoscience and Mineral Resources, Daejeon 34123, South Korea

^6^ Department of Earth System Sciences, Yonsei University, Seoul 03722, South Korea

**Contents**

1) Supplementary tables

2) Character statements

3) Data matrix

4) Supplementary references

**1. Supplementary tables**

**Supplementary Table 1.** Measurements (mm) of the skull of *Tarchia tumanovae* sp. nov. (MPC-D 100/1353). The asterisk refers to incomplete measurement due to damage.

| Element |  | | Measurements |
| --- | --- | --- | --- |
| Skull dimension | Length | Rostral tip to squamosal horns | 423.1 |
|  |  | Rostral tip to occipital condyle | 402.4 |
|  | Width | Across supraorbitals | 350.2 |
|  |  | Across squamosal horns | 382.8 |
|  |  | Across quadratojugal horns | 418.6* |
| Orbit dimension | Width | | 60.6 |
|  | Height | | 64.4 |

**Supplementary Table 2.** Measurements (mm) of dorsal, dorsosacral, parasacral, sacral, and caudosacral vertebrae of *Tarchia tumanovae* sp. nov. (MPC-D 100/1353). The asterisk refers to incomplete measurement due to damage.

| Element | Centrum  length | Anterior surface of the centrum | | Posterior surface of the centrum | | Total vertebral height |
| --- | --- | --- | --- | --- | --- | --- |
|  |  | Width | Height | Width | Height |  |
| 4^th^ dorsal | 117.4 | 120 | 121.7 | 120.9 | 126.9 | 366.9 |
| 11^th^ dorsal | 115.2 | 108.9 | 116.6 | 113.1 | 108 | 339.6 |
| 1^st^ dorsosacral | 133.2 | 112.3 | 116.3 | 96.4 | 107.4 | 252.5* |
| 2^nd^ dorsosacral | 114.3 | 96.4 | 107.4 | 89.5 | 98.4 | 287.3* |
| 3^rd^ dorsosacral | 119.3 | 89.5 | 98.4 | 98.4 | 83.5 | 340 |
| parasacral | 95.4 | 98.4 | 83.5 | 86.5 | 70.6 | 337 |
| 1^st^ sacral | 102.4 | 86.5 | 70.6 | 82.5 | 76.5 | 323.1 |
| 2^nd^ sacral | 89.5 | 82.5 | 76.5 | 74.6 | 86.5 | 308.2 |
| 3^rd^ sacral | 84.5 | 74.6 | 86.5 | 107.4 | 95.4 | 304.2 |
| 1^st^ caudosacral | 129.2 | 107.4 | 95.4 | 112.3 | 92.4 | 294.2* |
| 2^nd^ caudosacral | 93 | 123.5 | 112.4 | 115.5 | 103.8 | 296.9 |

**Supplementary Table 3.** Measurements (mm) of the tail club of *Tarchia tumanovae* sp. nov. (MPC-D 100/1353). The asterisk refers to incomplete measurement due to damage.

| Element |  | Measurements |
| --- | --- | --- |
| Tail club handle length | Length | 1571.1 |
| Tail club knob | Maximum width | 549.5 |
|  | Maximum length | 470.1 |
|  | Maximum height | 170.3 |

**Supplementary Table 4.** Measurements (mm) of ilia of *Tarchia tumanovae* sp. nov. (MPC-D 100/1353). The asterisk refers to incomplete measurement due to damage.

| Element |  | Measurements |
| --- | --- | --- |
| Right ilium | Length | 930.9 |
|  | Length of preacetabular process | 685.6 |
|  | Length of postacetabular process | 188.7 |
| Left ilium | Length | 919.6 |
|  | Length of preacetabular process | 680.4 |
|  | Length of postacetabular process | 201 |
| Left ischium | Length | 17.2 |
|  | Proximal width | 22.6 |

**2. Character statements**

Below is the character from Penkalski and Tumanova^1^, which is modified in this study.

Character 5. Remodeled squamosal horns

0: absent

1: present

Below are the two new characters that are added in this study.

Character 22. Skull in lateral view

0: quadrate-quadratojugal region normal

1: quadrate-quadratojugal region anteriorly situated

Character 23. Skull in occipital view

0: interpterygoid vacuity non-visible

1: interpterygoid vacuity visible

**3. Data matrix**

#NEXUS

BEGIN DATA;

DIMENSIONS NTAX=7 NCHAR=23;

FORMAT GAP = - MISSING = ? SYMBOLS = "0 1 2";

MATRIX

Pinacosaurus_grangeri 01?11?01001?01?10000?00

INBR_21004 01211011?00111010010100

MPC_D100/1338 012111010011??01?1?110?

MPC_100/151 10000100011000000101111

MPC-D_100/1353 11121?11111000101011011

PIN_3142/250 11121?11121000101011000

ZPAL_MgD_I/111 11121??11210??1011?1???

;

END;

**4. Supplementary references**

1. Penkalski, P. & Tumanova, T. The cranial morphology and taxonomic status of *Tarchia* (Dinosauria: Ankylosauridae) from the Upper Cretaceous of Mongolia. *Cretac. Res.* **70**, 117–127 (2017).
